# Hypomethylation‐associated Sox11 upregulation promotes oncogenesis via the PI3K/AKT pathway in OLP‐associated OSCC

**DOI:** 10.1111/jcmm.18556

**Published:** 2024-07-22

**Authors:** Yi Liu, Peilin Cao, Li Xiao, Na Tang, Wei Fei, Xue Li

**Affiliations:** ^1^ Department of Stomatology, Sichuan Provincial People's Hospital University of Electronic Science and Technology of China Chengdu Sichuan China; ^2^ Sichuan Provincial Key Laboratory for Human Disease Gene Study, Center for Medical Genetics, Department of Laboratory Medicine, Sichuan Provincial People's Hospital, School of Medicine University of Electronic Science and Technology of China Chengdu Sichuan China; ^3^ Department of Stomatology Sichuan Provincial People's Hospital Wenjiang Hospital Chengdu China

**Keywords:** DNA hypomethylation, OLP‐associated OSCC, PI3K/AKT pathway, Sox11, Warburg effect

## Abstract

Oral lichen planus (OLP) is a particularly prevalent oral disorder with the potential to progress to oral squamous cell carcinoma (OSCC). SRY‐box transcription factor 11 (Sox11) has been reported to serve as a prognostic marker for various cancers. However, the role and mechanism of Sox11 in OLP‐related OSCC are unknown. Our results indicated that Sox11 was highly expressed, and that Sox11 promoter methylation was significantly reduced in OLP‐associated OSCC tissues. High Sox11 expression and Sox11 promoter hypomethylation indicate a poor patient prognosis. According to in vivo and in vitro experiments, the knockdown of Sox11 inhibited proliferation, invasion, and migration while driving its apoptotic death in OSSC cells; Sox11 overexpression exerted the opposite effect as Sox11 knockdown. Mechanistically, knockdown of Sox11 inhibited PI3K/AKT and glycolysis pathway, and overexpression of Sox11 enhanced the PI3K/AKT and glycolysis pathways in OSCC cells. In addition, we demonstrated that Sox11 overexpression accelerated the progression of OSCC, at least in part by promoting PI3K/AKT pathway activation. In conclusion, our data indicated that the DNA hypomethylation‐associated upregulation of Sox11 could promote oncogenic transformation via the PI3K/AKT pathway in OLP‐associated OSCC. Therefore, Sox11 might be a reliable biomarker for predicting the progression of precancerous oral tissues.

## 1 INTRODUCTION

Oral cancers include malignant neoplasms that develop in the oral cavity or lip. In 2022 alone, there are forecast to be 54,000 new oral cancer diagnoses and 11,230 deaths throughout the world.^1^ While there have been some advances in diagnosing and treating oral cancer, patients still face a 5‐year survival of under 50% and a ~65% recurrence rate.^2^ Oral squamous cell carcinoma (OSCC) is a primary subtype of oral cancer.^3^ Many OSCC patients experience poor prognostic outcomes and high recurrence rates in part because the disease is often only diagnosed when in a relatively advanced state.

Further research exploring the aetiology of OSCC is critical to mitigating associated morbidity and mortality. OSCC tumours can arise from various potentially malignant oral disorders, including oral lichen planus (OLP), which is highly prevalent and associated with rates of malignancy that are generally thought to be underestimated.^4^, ^5^ As a chronic mucocutaneous condition with a range of clinical manifestations, OLP has the potential to progress to OSCC through poorly defined mechanisms.^6^ As such, more work is necessary to elucidate the process of OSCC development from OLP to provide patients with timely and effective treatment.

OLP is a chronic inflammatory oral condition that primarily impacts the tongue, gingiva, and buccal mucosa, resulting in symptoms such as blisters, erosions, erythema, white papules, striations, and plaques.^7^ OLP is classified as a premalignant disorder by the WHO, with the exact risk of transformation being poorly understood and suggesting a need for close patient monitoring. At the cellular level, OLP is associated with a chronic immune response mediated by T cells and abnormal epithelial keratinization activity. However, the specific molecular aetiology of OLP and the factors that ultimately drive it to progress to OSCC have yet to be established. Further studies exploring protein‐level and genetic changes in OLP‐associated OSCC are thus warranted to improve the current understanding of the malignant potential of OLP such that OLP‐associated OSCC can be more readily diagnosed and treated at an early stage.

The human SOX transcription factor family comprises over 20 proteins.^8^ Of these, the SOXC family member Sox11 is primarily expressed in the context of embryogenesis, whereas its expression is generally undetectable in differentiated adult tissues. Sox11 regulates stem and progenitor cell behaviours, often acting with SOX12 and SOX4 to regulate critical processes such as skeletal tissue development and neurogenesis.^9^, ^10^, ^11^ Sox11 has also been shown to drive mantle cell lymphoma (MCL) progression,^12^, ^13^ with other studies have shown it to play a similar oncogenic role in melanoma and breast cancer.^14^, ^15^ Sox11 overexpression has also been reported in small cell lung cancer^16^ Burkett's lymphoma, and leukaemia.^17^ Contradictory evidence about the role and expression of Sox11 in glioma, MCL, and ovarian cancer has, however, been reported.^18^, ^19^, ^20^ As such, Sox11 may play a tissue‐ and context‐specific role in the progression of different tumours. While Sox11 is thus a critical regulator of the onset and progression of cancer, its specific functional relevance in the progression of OLP to OSCC has yet to be established.

DNA hypomethylation is an epigenetic change that involves a reduction in the level of DNA methylation, which is particularly common in some types of cancer.^21^ Sox11 expression is associated with DNA methylation levels in some cancers.^22^, ^23^ The study found that Sox11 expression in mantle cell lymphoma is associated with DNA methylation levels, which can inhibit tumour cell proliferation.^24^ The methylation rate of the Sox11 gene was higher in cervical cancer tissues, while its mRNA expression was much lower than that in normal cervical tissues.^25^ The DNA methylation level of Sox11 was relatively high in hepatocellular carcinoma.^26^, ^27^ Therefore, low expression of Sox11 may have an association with DNA methylation in cancer progression. Thus, the present study further explored whether Sox11 expression was associated with DNA hypomethylation in OSCC.

The Warburg effect refers to the phenomenon that tumour cells tend to acquire energy through glycolysis rather than oxidative phosphorylation, even under oxygen‐sufficient conditions. Warburg effect is associated with cell migration and invasion. Cancer cells have the character that they tend to generate adenosine triphosphate (ATP) through aerobic glycolysis no matter whether there is enough oxygen or not.^28^, ^29^ Some reports indicate that glycolysis can lower PH value in the tumour microenvironment and subsequently influence signal transduction.^30^ Inhibition of glucose transporters, such as glucose transporter 4 (GLUT4), would significantly suppress the invasion or migration of cancer cells. However, the role of Sox11 on the Warburg effect, proliferation, apoptosis, and invasion of OSCC cells remains unknown. We have investigated the function and underlying mechanism of Sox11 in OSCC. The present study was therefore developed to assess differences in Sox11 expression in OLP‐associated OSCC and OLP tissues and clarify the function of Sox11 as a modulator of OSCC cell proliferative, migratory, and invasive activity.

## 2 MATERIALS AND METHODS

A detailed description was provided in the Supplementary materials.

### 2.1 Statistical analysis

GraphPad Prism 9.0 (GraphPad, CA, USA) was used to analyse all data reported as means ± standard deviation (SD). Results were assessed using Student's *t*‐tests and one‐way ANOVAs, while chi‐square tests were employed to examine the relationship between Sox11 expression levels and patient clinicopathological characteristics. The median Sox11 expression level was used to stratify patients into the low‐ and high‐expression groups. Survival analyses were performed using Kaplan–Meier curves and the log‐rank test. *P* < 0.05 was the threshold of significance.

## 3 RESULTS

### 3.1 Sox11 is upregulated in OLP‐associated OSCC tissues, and high expression of Sox11 is associated with poor prognosis in OSCC patients

**FIGURE 1:**
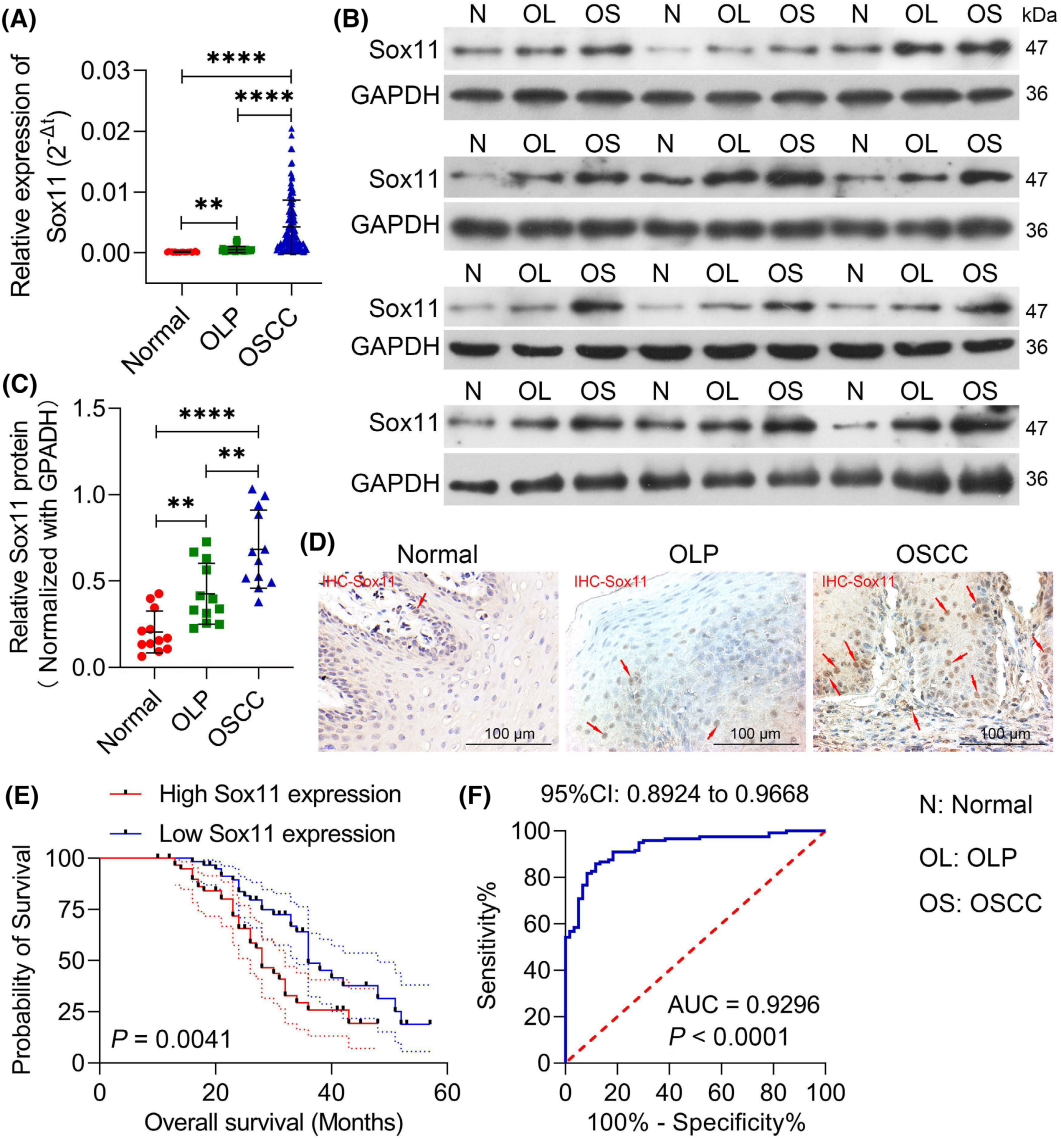
The upregulation of Sox11 in OLP‐associated OSCC is related to poorer prognostic outcomes. (A) qPCR analysis revealed the upregulation of Sox11 expression in OSCC tissues compared to OLP and normal oral mucosal tissue samples (Normal: *n* = 20; OLP: *n* = 40; OSCC: *n* = 120). (B) Sox11 level was assessed by western blot in normal oral mucosa tissue (N), oral lichen planus (OL) and oral squamous cell carcinoma (OS) tissue samples (*n* = 12). (C) Quantitative analysis of Sox11 protein in the western blotting results. (D) IHC staining was adopted to confirm the Sox11 expression in normal oral mucosa tissue, OLP and OSCC tissue samples (E) The relationship between Sox11 level and OSCC patient overall survival was assessed through a Kaplan–Meier analysis, and the cut‐off point (Median) was 0.002577 to distinguish the high and low expression of Sox11. (F) ROC curves were employed to assess the association between Sox11 level in OSCC and normal oral mucosa tissue samples. Data are shown in means ± SD. **p < 0.01, ****p < 0.0001. (×200, scale bars, 100 μm).

Sox11, a member of the SOX family, plays a regulatory role in specific biological processes.^31^ Multiple studies demonstrated that the aberrant expression of Sox11 is related to cancer progression and prognosis.^16^, ^32^, ^33^ To investigate the role of Sox11 in OLP‐associated OSCC progression, we first evaluated the expression of Sox11 in normal oral mucosa (*n* = 20), OLP (*n* = 40), and OSCC tissues (*n* = 120). Fortunately, we found that Sox11 expression was significantly elevated in OLP tissues relative to normal oral mucosa tissue samples, with further Sox11 upregulation in OSCC tissues relative to OLP tissues (Figure 1A). Increases in Sox11 expression were further confirmed through Western blot analyses for these three sample types (*n* = 12/group), confirming significant Sox11 upregulation in OSCC tissues compared to both normal tissues and OLP samples and 12 cases of OSCC tissues developed from OLP tissues (Figure 1B,C). Next, IHC was further adopted to confirm the expression of Sox11 (yellow‐brown granules) in clinical tissue samples, revealing more pronounced Sox11 staining intensity in OLP‐associated OSCC tissue samples (OLP tissue develops into OSCC tissue, and OLP tissue is a precancerous lesion of OSCC tissue) relative to OLP tissue samples (Figures 1D and S1–S3). These data suggest that Sox11 may be an oncogene in OLP‐associated OSCC progression. Besides, the data indicated that higher levels of Sox11 expression were found to be significantly related to the TNM stage (*p* = 0.0033), degree of tumour differentiation (*p* = 0.0176), and tumour size (*p* = 0.0005) in OSCC patients (Table 1). Sox11 levels were not significantly related to patient age or gender. The follow‐up data were used to conduct survival analyses of the patients included in this study, revealing that patients expressing high levels of Sox11 exhibited worse overall survival (OS) (Figure 1E). ROC curves further confirmed the potential diagnostic value of Sox11 in OSCC, revealing a high level of diagnostic sensitivity (AUC = 0.9296, Figure 1F). As such, these results suggested that Sox11 may offer value as a prognostic and diagnostic biomarker for OLP‐associated OSCC patients.

**TABLE 1:** Correlation between Sox11 expression and clinicopathological features in 120 cases of OSCC patients.

Factors	*N*	Sox11 expression (*n* = 120)		*p* value
		Low expression (*n* = 60)	High expression (*n* = 60)	
Age				
<60	59	27	32	0.7078
≥60	61	30	31	
Gender				
Male	57	25	32	0.3509
Female	63	33	30	
Tumour size (cm)				
<3	57	39	18	**0.0005**
≥3	63	23	40	
TNM stage				
I–II	58	38	20	**0.0033**
III–IV	62	24	38	
Differentiation				
Poor	59	23	36	**0.0176**
Well	61	37	24	

*Note*: Bold values presnted in table 1 were < 0.05.

Abbreviation: OSCC, oral squamous cell carcinoma.

### 3.2 Hypomethylation of the Sox11 promoter predicts a poorer OLP‐associated OSCC patient prognosis

**FIGURE 2:**
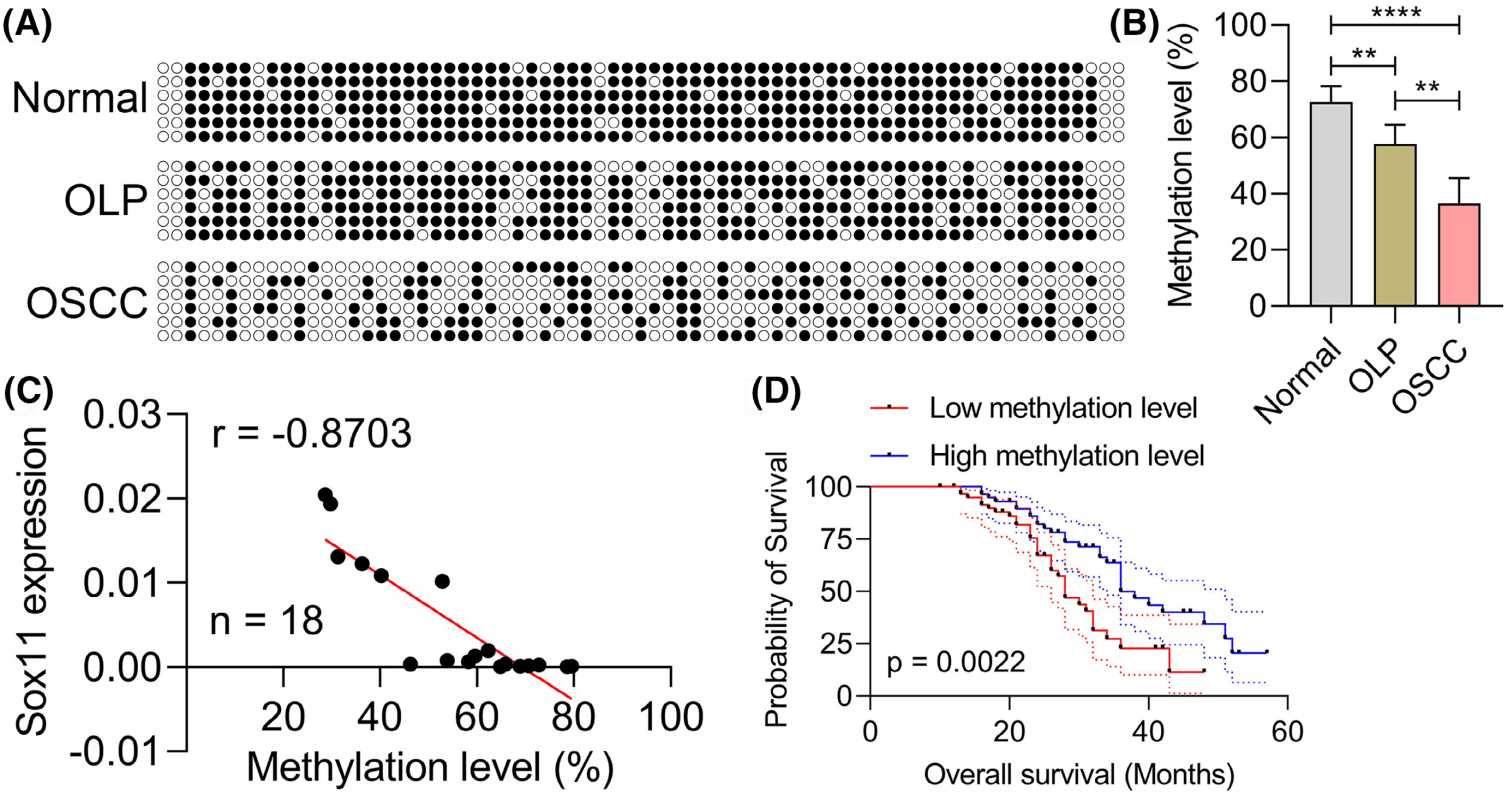
Sox11 promoter hypomethylation is linked to poorer prognostic outcomes in OLP‐associated OSCC. (A) Sox11 CpG methylation was analysed via BSP in normal, OLP and OSCC tissues. ● methylated CpG; ○ unmethylated CpG. (B) Quantification of methylation levels. (C) Correlation between Sox11 expression and Sox11 promoter methylation status. (D) The relationship between patient overall survival and Sox11 promoter methylation was assessed using Kaplan–Meier curves. Data are shown in means ± SD. **p < 0.01, ****p < 0.0001.

It is reported that Sox11 expression is related to DNA methylation status.^22^, ^34^ The methylation status of the Sox11 gene is also relevant to the prognosis and progression of tumours, which can be used as a useful biomarker for tumour diagnosis and prognosis evaluation.^22^, ^23^, ^35^ Thus, we further explore the level and prognosis of Sox11 promoter methylation in OSCC tissues of patients. BSP was conducted to analyse the methylation status of the Sox11 promoter region. Relative to normal paracancerous tissue samples, both OSCC and OLP tissues exhibited significant reductions in the levels of Sox11 promoter methylation, with respective methylation rates in average, OLP, and OSCC tissues of 72.59 ± 5.69, 57.70 ± 6.89, and 36.50 ± 9.12, respectively (Figure 2A,B). Sox11 expression was also negatively correlated with the levels of Sox11 promoter methylation in OSCC tissue samples (Figure 2C). When OSCC samples (*n* = 120) were stratified into two groups based on whether their Sox11 promoter methylation levels were below or above the median value, patients exhibiting lower levels of Sox11 promoter methylation were found to exhibit a reduced OS rate (Figure 2D). Together, these data support a model wherein the hypomethylation of the Sox11 promoter is linked to a poorer prognosis in patients diagnosed with OLP‐associated OSCC.

### 3.3 DNA methylation regulates Sox11 expression in OLP‐associated OSCC

**FIGURE 3:**
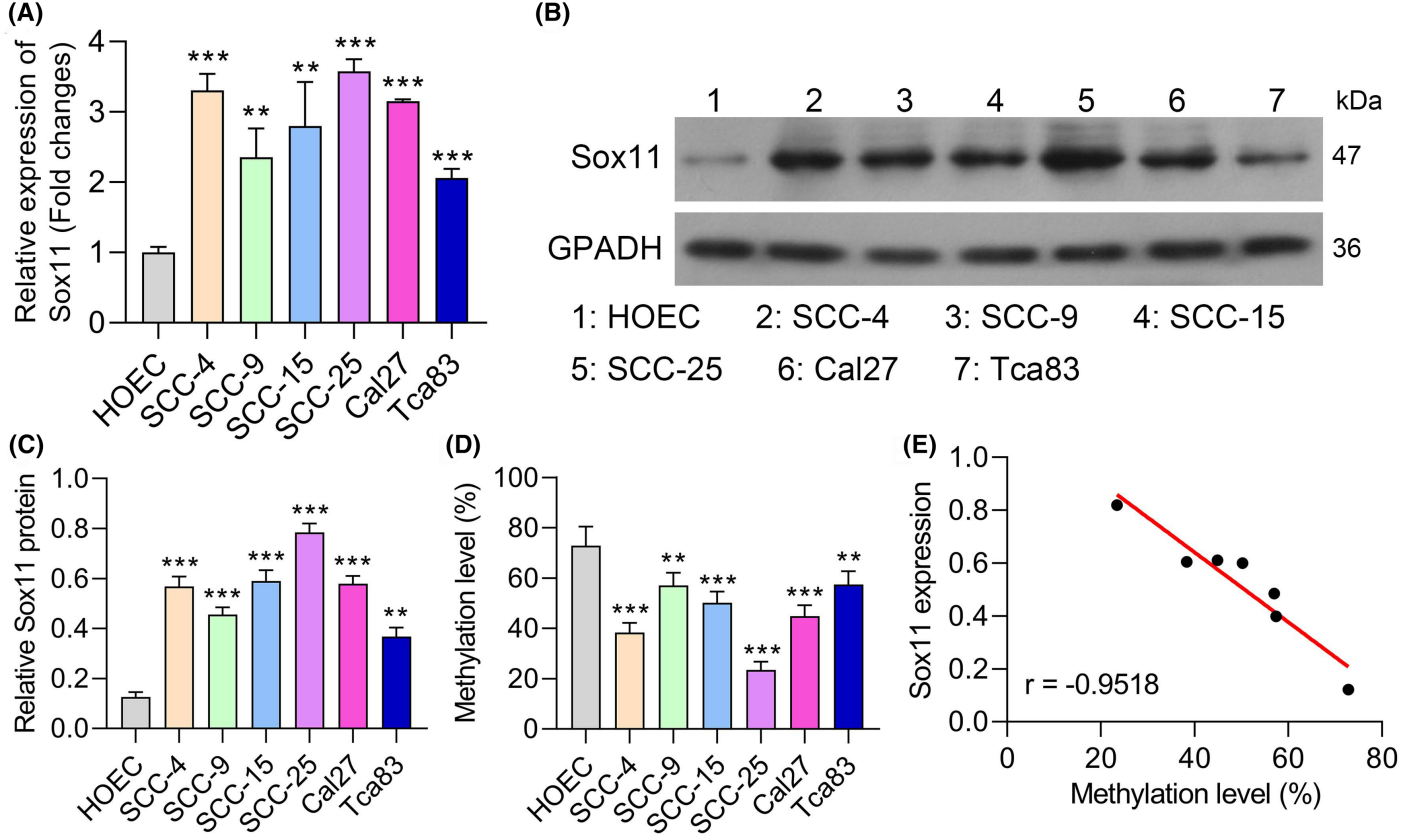
DNA methylation regulates Sox11 expression in OLP‐associated OSCC. (A–C) Sox11 expression was tested by RT‐qPCR and western blot in oral epithelial cells, the analysed OSCC cell lines (SCC‐4, SCC‐9, SCC15, SCC25, CAL27, and Tca83). (D) CpG methylation was assessed via BSP in OSCC cell lines and oral mucosal HOEC cells. (E) The correlation between Sox11 expression and the level of Sox11 promoter methylation was assessed using the correlation analysis in OSCC cell lines. Data are shown in means ± SD. **p < 0.01, ***p < 0.001.

To extend the above findings in an in vitro setting, Sox11 expression levels were next quantified in OSCC cell lines and control HOEC cells via qPCR, revealing significantly increased *Sox11* mRNA levels in analysed OSCC cells (Figure 3A). Western blot similarly confirmed the upregulation of Sox11 in OSCC cells compared to HOEC cells (Figure 3B,C). In line with the human data presented in the previous section, the six tested OSCC cell lines exhibited reduced Sox11 promoter methylation compared to HOEC cells (Figure 3D). Sox11 expression was also negatively correlated with Sox11 promoter methylation levels in these OSCC cell lines (Figure 3E). These data suggest that DNA hypomethylation similarly contributes to Sox11 overexpression in OSCC cells in vitro.

### 3.4 Sox11 promotes OSCC cell proliferation and prevents apoptotic cell death

**FIGURE 4:**
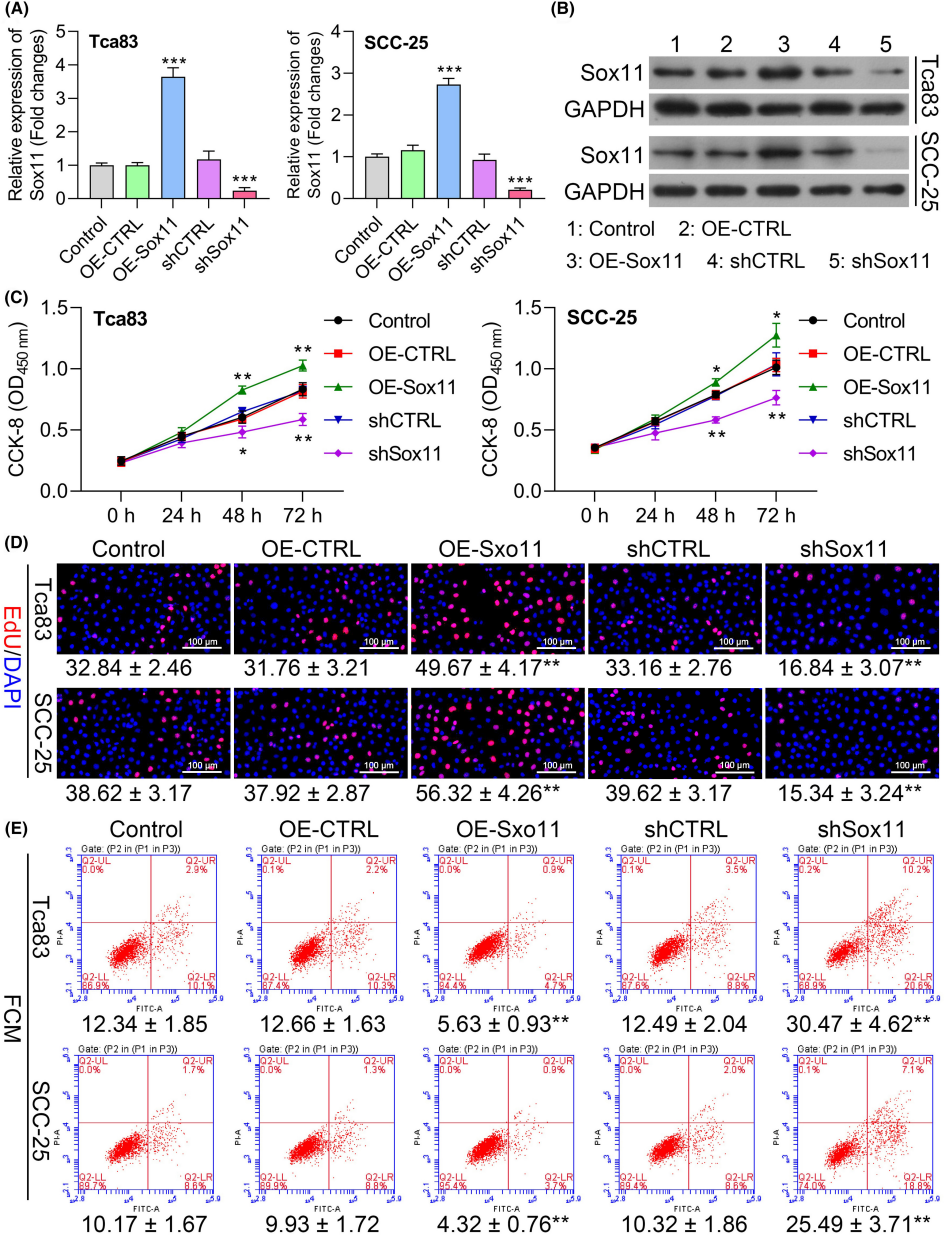
Sox11 enhances proliferation and protects against apoptotic death in OSCC cells. (A, B) Sox11 knockdown and overexpression efficiencies were assessed via qPCR and western blot in SCC‐25 and Tca83 cells following shSox11 or OE‐Sox11 transfection. (C, D) CCK‐8 and EdU assays were employed to assess the proliferation of SCC‐25 and Tca83 cells after transfection. (E) Apoptosis in transfected SCC‐25 and Tca83 cells was evaluated by flow cytometry. Data are shown in means ± SD. *p < 0.05; **p < 0.01; ***p < 0.001. (×200, scale bars, 100 μm).

As the SCC‐25 and Tca83 cell lines exhibited the highest and lowest levels of Sox11 expression at the mRNA and protein levels among tested cell lines, these cells were used in subsequent assays. Sox11 was knocked down in these cell lines using a shSox11 construct (Figure 4A,B), while an overexpression plasmid was used to overexpress Sox11 in both SCC‐25 and Tca83 cells, as confirmed via qPCR and Western blotting (Figure 4A,B). Subsequent CCK‐8 assays revealed that while overexpressing Sox11 significantly increased the viability of these OSCC cells, Sox11 knockdown had the opposite impact (Figure 4C). An EdU incorporation assay further revealed markedly enhanced proliferation in cells overexpressing Sox11 compared to negative control cells, and cells in which Sox11 had been knocked down grew less efficiently than control cells transfected with a shCTRL construct (Figure 4D). OSCC cell apoptosis was enhanced when Sox11 was knocked down, whereas the overexpression of Sox11 protected these cells against undergoing apoptotic death (Figures 4E and S2). Together, these findings thus indicated that Sox11 can stimulate cellular proliferation and prevent the apoptotic death of OSCC cells.

### 3.5 Sox11 enhances glycolysis and promotes OSCC cell migration and invasion

**FIGURE 5:**
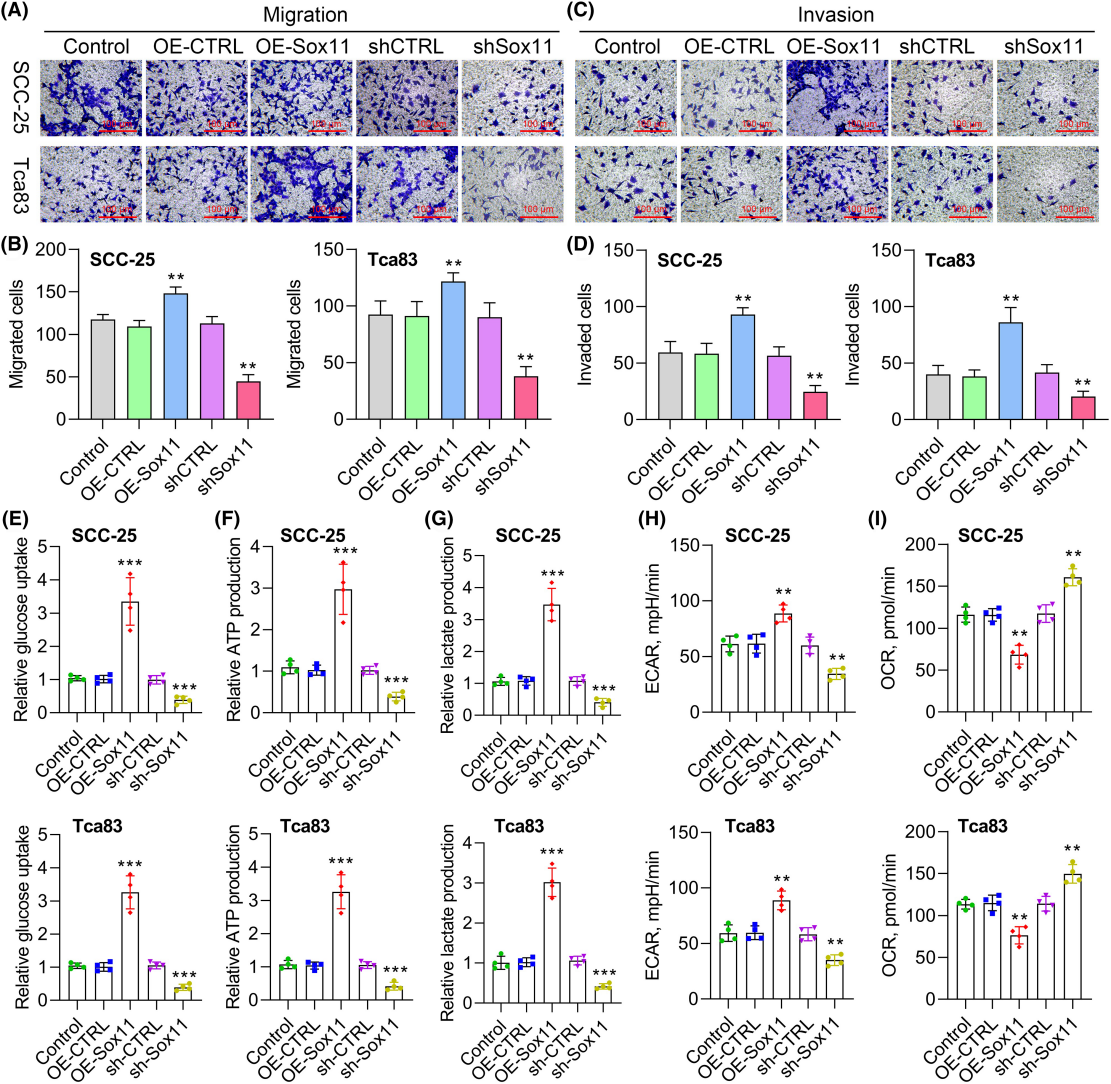
Sox11 drives the enhanced invasion, migration and glycolysis in OSCC cells. (A, B) Cell migration was confirmed using Transwell assay in Sox11‐overexpressed or silenced SCC‐25 and Tca83 cells. (C, D) Cell invasion was confirmed using Transwell assay in Sox11‐overexpressed or silenced SCC‐25 and Tca83 cells. Glucose uptake (E), ATP production (F) and lactate production (G) were tested by glucose uptake, lactate production and intracellular ATP assays in Sox11‐overexpressed or silenced SCC‐25 and Tca83 cells. (H) The Seahorse XF extracellular flux analyser denoted the change in the ECAR rate in SCC‐25 and Tca83 cells. (I) The Seahorse XF extracellular flux analyser determined the rate of OCR in SCC‐25 and Tca83 cells. Data are shown in means ± SD. **p < 0.01; ***p < 0.001. (×200, scale bars, 100 μm).

Next, a Transwell assay system assessed OSCC cell migratory and invasive activity. When Sox11 was overexpressed, the migration and invasion of cells were enhanced, whereas Sox11 knockdown had the opposite impact on these phenotypes (Figure 5A–D). These data thus provide further support for the role of Sox11 as an oncogenic driver of OSCC malignancy. It is reported that the Warburg effect can promote the migration and invasion of tumour cells through multiple mechanisms.^36^ The accumulation of lactate produced by tumour cells through aerobic glycolysis leads to acidification of the tumour microenvironment and accelerates tumour cell migration and invasion.^37^, ^38^ Lactate can also promote new angiogenesis and create an environment for tumour cell migration.^39^ Besides, the Warburg effect can transform polar epithelial cells into motile stromal cells, thereby acquiring the ability to migrate.^39^, ^40^ Therefore, we further explored the effect of Sox11 on the Warburg effect of OSCC cells. The results first revealed that Sox11 overexpression dramatically enhanced glucose uptake and lactate production, and Sox11 knockdown inhibited glucose uptake and lactate production in OSCC cells (Figure 5E,G). Consistently, ATP level was increased in Sox11 OE SCC‐25 and Tca83 cells and further reversed by knockdown of Sox11 (Figure 5F). To confirm whether or not Sox11 is associated with metabolism type in the tumour cells, we checked ECAR and OCR in tumour cells (Figure 5H,I). The glycolytic capacity and steady state glycolysis flux (Figure 5H), ATP production, and basal respiration (Figure 5I) were significantly suppressed in Sox11 downregulated OSCC cells, indicating that Sox11 can promote glycolysis flux. This implied that Sox11 is associated with enhanced cell migration and invasion and increased glycolytic rate in OSCC cell lines.

### 3.6 Sox11 drives OSCC cell proliferation through PI3K/AKT signalling activity

**FIGURE 6:**
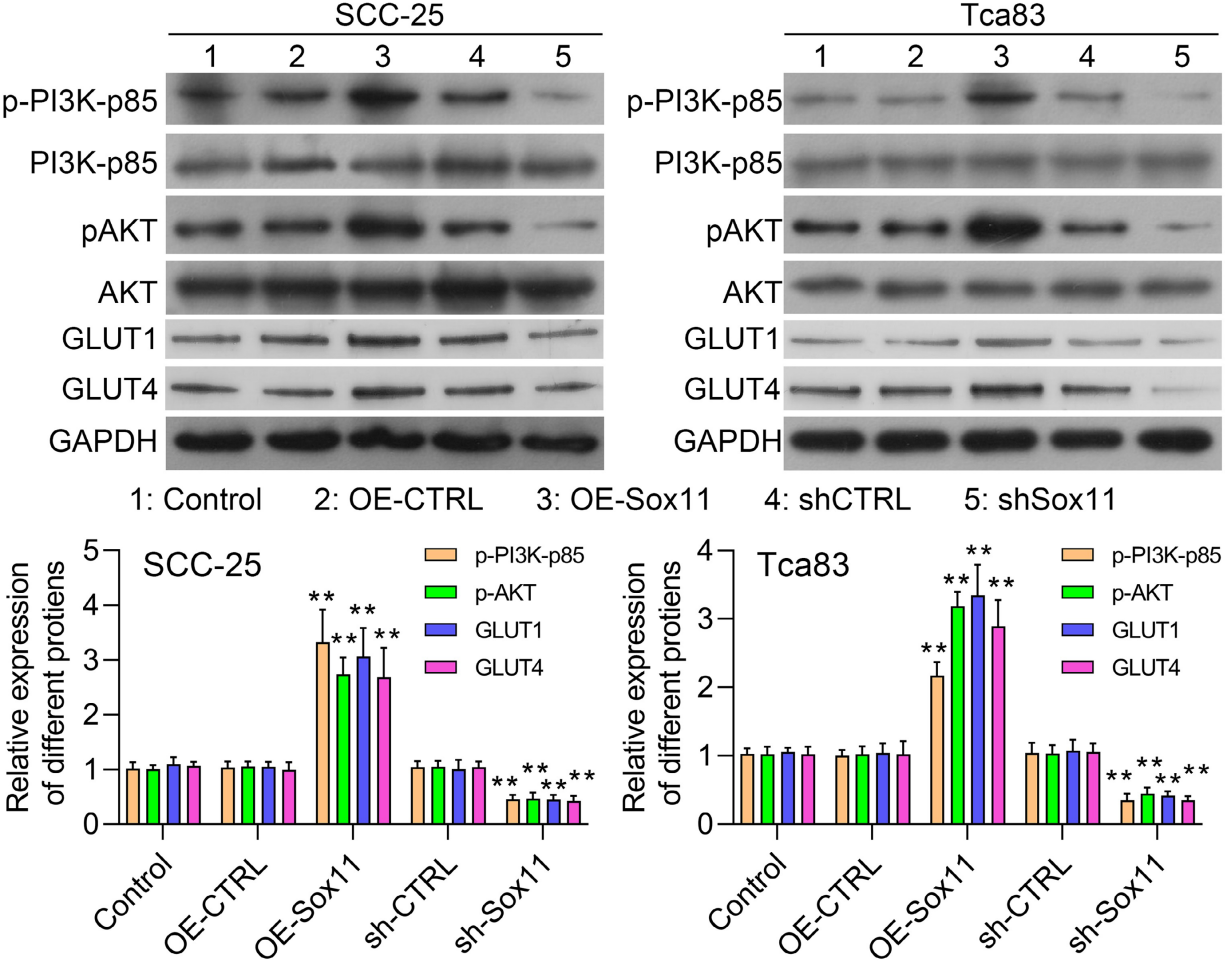
The effect of Sox11 on the PI3K/AKT pathway and glycolysis‐related proteins. p‐PI3K‐p85, PI3K‐p85, p‐AKT, AKT, GLUT1 and GLUT4 expression levels were measured via western blot from the SCC‐25 and Tca83 cells in five treatment groups (control, OE‐CTRL, OE‐Sox11, shCTRL and shSox11). Data are representative of experiments repeated in triplicate. Data are shown in means ± SD. **p < 0.01.

Based on the hypothesis that Sox11 may promote OSCC proliferation in part via modulating the PI3K/AKT pathway, p‐PI3K‐p85 and p‐AKT expression levels in OSCC cells were evaluated by western blot. While p‐PI3K‐p85 and p‐AKT levels were reduced by Sox11 knockdown, this effect was reversed following Sox11 overexpression (Figure 6). Thus, Sox11 promoted the progression of OSCC, at least in part, via activating the PI3K/AKT signalling.

### 3.7 PI3K/AKT inhibitors suppress the promoting effect of Sox11 overexpression in OSCC cells

**FIGURE 7:**
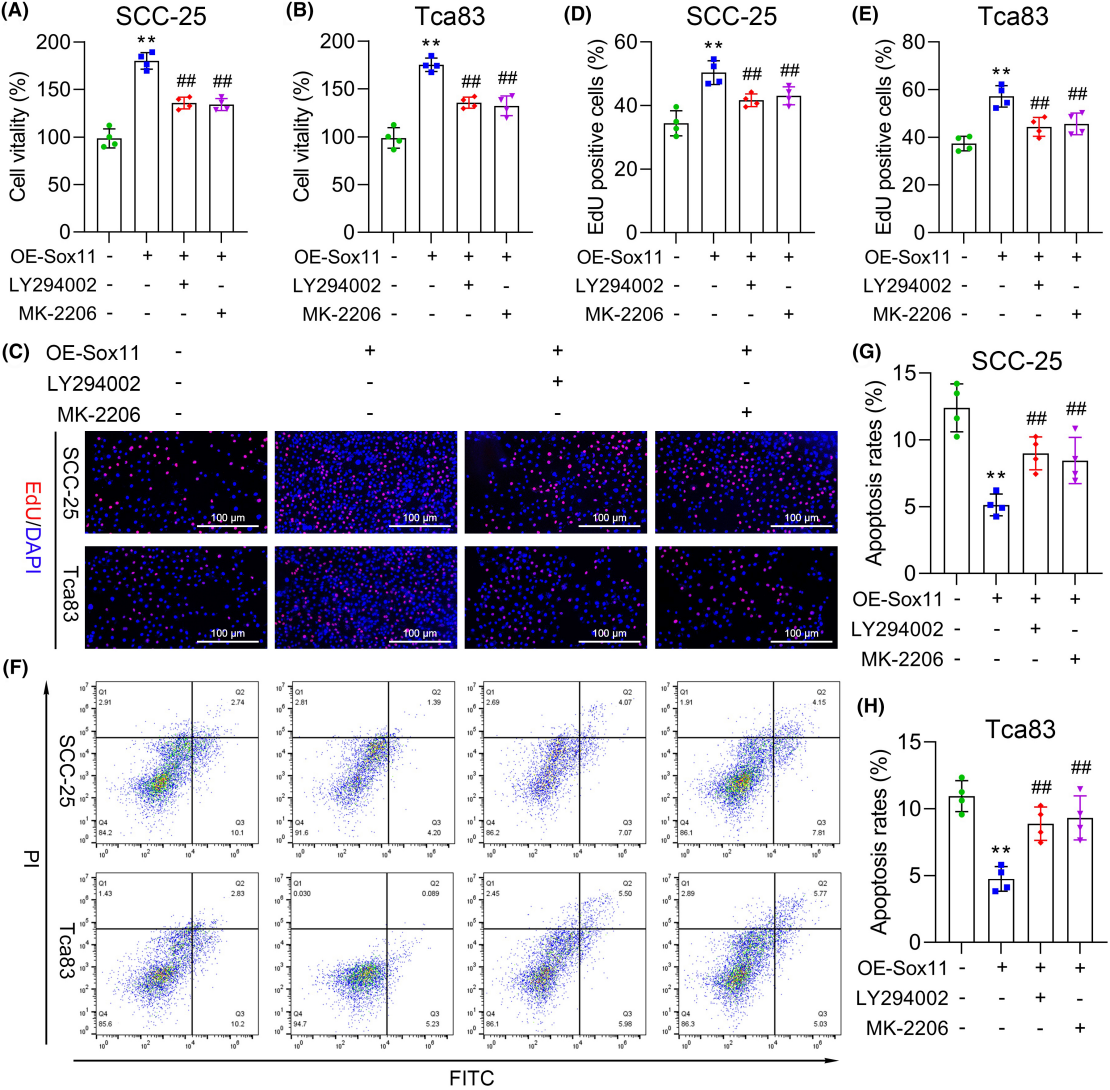
PI3K/AKT inhibitors reverse the Sox11 overexpression‐mediated cell viability promotion and apoptosis inhibition in SCC‐25 and Tca83 cells. Cell proliferation was determined by CCK‐8 (A, B) and EdU assays (C–E) in Sox11‐overexpressed SCC‐25 and Tca83 cells with or without PI3K/AKT inhibitor LY294002 or MK‐2206. (F–H) Flow cytometry analysis of apoptosis in the treated SCC‐25 and Tca83 cells. Data are shown in means ± SD. **p < 0.01 compared with the control group; ^##^ *p* < 0.01 compared with the Sox11 overexpression group. (×200, scale bars, 100 μm).

LY294002 and MK‐2206 are specific inhibitors of PI3K and AKT, respectively.^41^ Here, we checked if inhibition of PI3K/AKT by LY294002 or MK‐2206 is toxic to OSCC cells and whether or not the function of the inhibitor suppresses the Sox11‐mediated OSCC progression process. After Sox11 upregulation, proliferation in SCC‐25 and Tca83 cells increased significantly. However, such an increase was markedly retarded by LY294002 or MK‐2206 treatment (Figure 7A–E). Next, we explored whether Sox11 also functions in OSCC cell apoptosis via PI3K/AKT signalling. Flow cytometry analysis indicated that overexpression of Sox11 hindered the cell apoptosis of SCC‐25 and Tca83 cells, and applying the treatment of LY294002 or MK‐2206 (Figure 7F–H) can restore the result mentioned above. Thus, these results indicate that PI3K/AKT inhibitors reduced cell viability and induced apoptosis of SCC‐25 and Tca83 cells induced by upregulation of Sox11.

### 3.8 PI3K/AKT signalling plays a crucial role in Sox11‐stimulated tumorigenesis of OSCC

**FIGURE 8:**
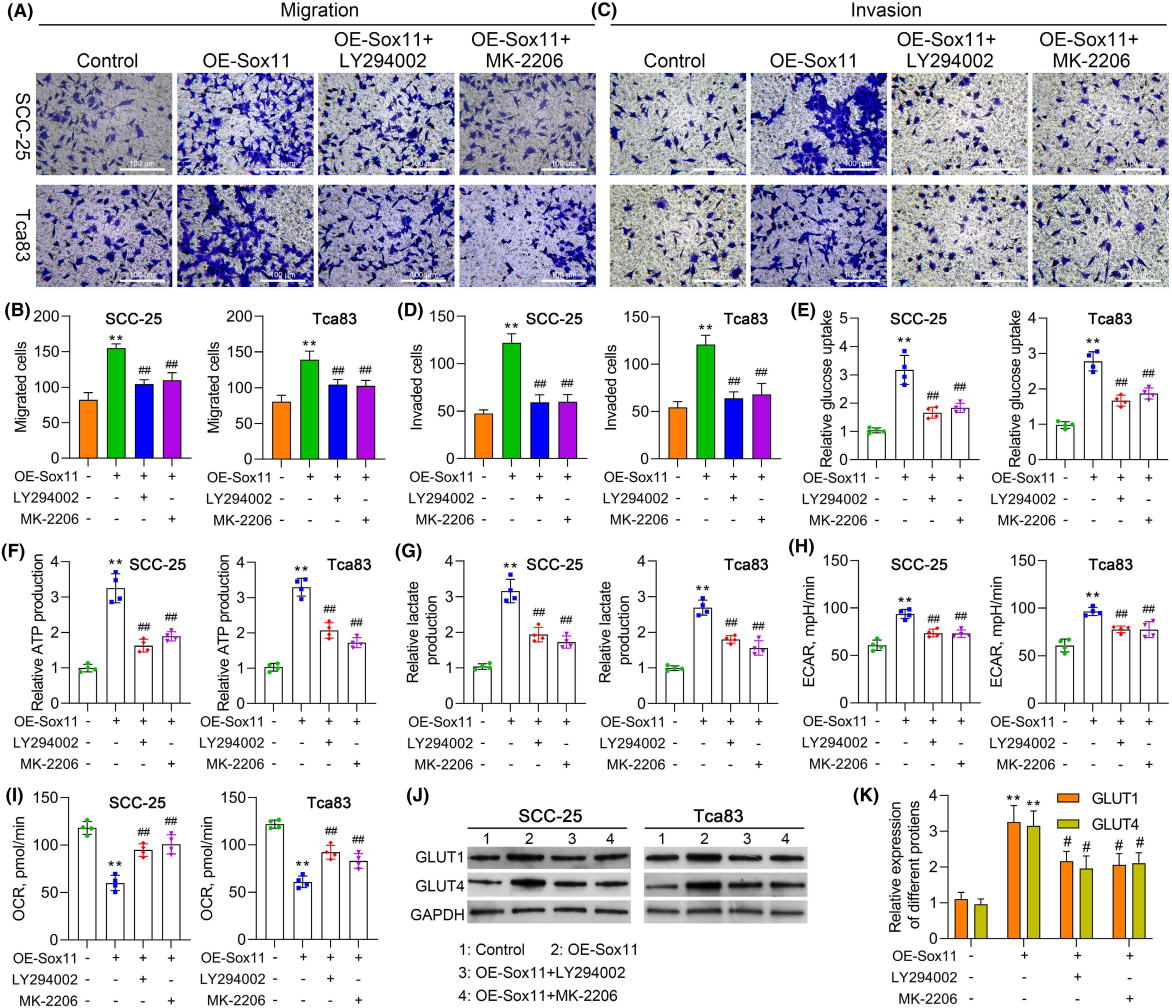
PI3K/AKT signalling plays a key role in Sox11‐stimulated tumorigenesis of OSCC. (A–D) Cell migration and invasion were determined by transwell assay in Sox11 OE SCC‐25 (A,B) and Tca83 cells (C,D) with or without further treatment of LY294002 or MK‐2206. (E–I) SCC‐25 and Tca83 cells were overexpressed for Sox11 and further treated with LY294002 or MK‐2206, followed by determination of glucose uptake (E), ATP production (F), lactate production (G) as well as ECAR and OCR levels (H, I). (J, K) Western blotting analysis of glycolysis enzymes (GLUT1 and GLUT4) in Sox11‐overexpressed SCC‐25 and Tca83 cells with or without further treatment of LY294002 or MK‐2206. Data are shown in means ± SD. **p < 0.01 compared with the control group; ^#^ *p* < 0.05; ^##^ *p* < 0.01 compared with the Sox11 overexpression group. (×200, scale bars, 100 μm).

Next, we explored whether the PI3K/AKT signalling also influences cell migration, invasion, or glycolysis in cancer cells. The transwell assay indicated that Sox11 upregulation promoted the migration and invasion of SCC‐25 and Tca83 cells, and such an increase was weakened by further application of LY294002 or MK‐2206 treatment (Figure 8A–D). To confirm whether PI3K/AKT signalling is involved in Sox11 overexpression‐induced‐glycolytic flux increasement, LY294002 or MK‐2206 treatment was applied in Sox11 upregulation cells and detected glycolysis flux. The promotion effect of overexpressed Sox11 on glucose uptake (Figure 8E), ATP level (Figure 8F), and lactate production (Figure 8G), as well as ECAR (Figure 8H) and OCR levels (Figure 8I) in SCC‐25 and Tca83 cells was entirely abolished by LY294002 or MK‐2206 treatment. We also found an association between GLUT1 or GLUT4 expression and Sox11 or PI3K/AKT expression. Specifically, the protein levels of GLUT1 and GLUT4 were increased in Sox11 OE SCC‐25 and Tca83 cells, whereas downregulated by LY294002 or MK‐2206 treatment (Figure 8J,K), suggesting that Sox11 could tightly regulate PI3K/AKT signalling.

### 3.9 Overexpression of Sox11 accelerates the growth of tumours via the PI3K/AKT pathway

**FIGURE 9:**
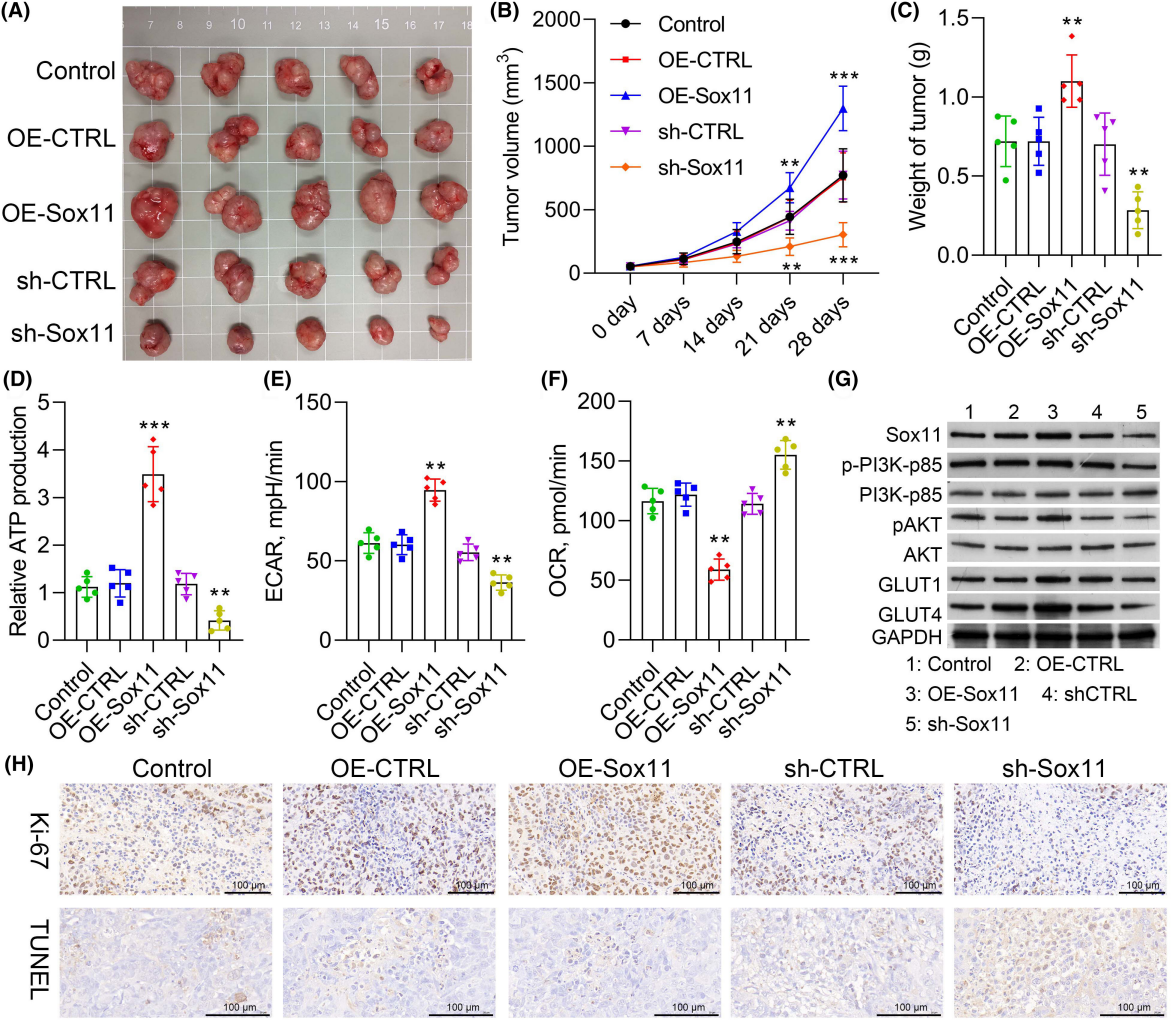
Overexpression of Sox11 accelerates the growth of tumours via the PI3K/ AKT pathway. (A) Tumour growth in Balb/c nude mice. Tumours in the control, OE‐CTRL, OE‐Sox11, sh‐CTRL, and sh‐Sox11 groups were collected at the end of the experiments. Tumour growth was assessed by tumour volume (B) and tumour weight (C) (*n* = 5). **p < 0.01; ***p < 0.001. (D) The relative level of ATP in the xenograft tissues was measured using an ATP assay. (E) The ECAR of the tumour. (F) The OCR of the tumour. (G) Expression levels of Sox11, p‐PI3K‐p85, PI3K‐p85, p‐AKT, AKT, GLUT1, and GLUT4 were evaluated by western blot. **p < 0.01, ***p < 0.001. (H) Representative images of Ki67 expression and TUNEL staining in CTRL, OE‐NC, OE‐Sox11, shCTRL and shSox11 cohorts, respectively. (×200, scale bars, 100 μm).

To confirm whether or not Sox11 and PI3K/AKT signalling promotes tumour progression in vivo, the OSCC cell with or without Sox11 upregulation or downregulation was injected into nude mice. Consistent with in vitro experiments, we found that the overexpression of Sox11 promotes tumour volume, and the downregulation of Sox11 suppresses tumour volume as shown in Figure 9A–C. Subsequently, the ATP assay of tumour tissue showed that Sox11 downregulation suppresses ATP generation, which was further reversed by overexpression of Sox11 (Figure 9D). Then, we analysed the ECAR and OCR of these tumour tissues; similarly, increased glycolysis flux mediated by Sox11 upregulation was entirely abolished by Sox11 knockdown (Figure 9E,F). Finally, a western blot was used to check the expression of p‐PI3K‐p85, p‐AKT, GLUT1, and GLUT4. The results were consistent with in vitro experiments (Figure 9G). Subsequently, IHC stains for xenograft tissue extracted from nude mice demonstrated that knock‐down within Sox11 can markedly downregulate Ki67, which is related to cancer growth (Figures 9H and S3A). Moreover, the TUNEL assay showed that Sox11 overexpression reduced apoptosis compared to control. However, apoptosis within OSCC tissues was markedly elevated within the Sox11 knock‐down cohort (sh‐Sox11) compared to the shCTRL cohort (Figures 9H and S3B).

## 4 DISCUSSION

Members of the SOX transcription factor family contain highly conserved HMG‐box DNA binding domains, which are composed to classify SOX family members into eight subgroups (A–H).^8^ The group C SOX protein SOX4 has previously been shown to promote the progression of OLP‐associated OSCC.^42^ Sox11 is a 441 amino acid group C SOX family member encoded on chromosome 2.p25.^43^ Prior work has suggested Sox11 as a critical oncogene in MCL.^44^ At the same time, in physiological settings, it is essential to develop the spinal cord, sympathetic neurons, sensory neurons, and several non‐neuronal tissue types.^45^ Sox11 additionally harbours a C‐terminal transactivation domain.^46^ Sox11 expression is mainly absent in adult tissues under normal conditions, although it is an essential mediator of the embryonic development of the limbs, kidneys, face, and neuronal tissues.^47^ Huang et al.^48^ recently established the ability of Sox11 to regulate SDCCAG8 and to promote thereby head and neck squamous cell carcinoma (HNSCC) progression. However, the functional role of Sox11 in OSCC has yet to be firmly established.

OSCC is among the most aggressive forms of head and neck cancer among humans,^49^ with affected patients often exhibiting a poor prognosis because the molecular mechanisms governing the oncogenic progression of oral precancerous states are not well understood such that many OSCC patients are only diagnosed when the disease is already relatively advanced.^50^ This underscores the importance of clarifying the molecular determinants of OSCC development and progression to establish biomarkers to aid in the early diagnosis of this deadly disease. These results indicate that Sox11 is upregulated in OLP‐associated OSCC relative to OLP, as the OSCC patients enrolled in this study developed OSCC following an initial diagnosis of OLP. Sox11 overexpression in OSCC cells further drove their rapid proliferation, migratory activity, and invasivity. Together, these data highlight Sox11 as an oncogenic driver of OLP‐associated OSCC and a potentially viable target for therapeutic intervention.

In the light of these promising data, the mechanisms underlying the ability of Sox11 to regulate the pathogenesis of OLP‐associated OSCC were explored in detail, with a particular focus on the processes of acetylation and methylation given their critical importance in several types of cancer.^51^ The hypermethylation of CpG residues within CpG island regions has been identified as an epigenetic driver of tumour suppressor gene inactivation in malignant cells,^52^ with multiple studies having confirmed the ability of DNA methylation to suppress tumour suppressor gene expression.^53^, ^54^ Research has also demonstrated that promoter hypomethylation can lead to oncogene activation. For example, promoter hypomethylation could cause the upregulation of matrix metalloproteinase‐1 (MMP1) in breast cancer^55^; promoter hypomethylation is related to upregulation of Syncytin‐1 in non‐small cell lung cancer^56^; hypomethylation could activate protein lin‐28 homolog B (LIN28B) to induce gastric cancer cell proliferation and metastasis.^57^ Accordingly, Sox11 promoter methylation status was analysed in tissues and cell lines, revealing the hypomethylation of this region in OSCC samples. In contrast, this region was hypermethylated in control tissues and cell lines. These results thus strongly suggest that the hypomethylation of the Sox11 promoter is a primary factor underlying its overexpression in OSCC.

Further, we found that the upregulation of Sox11 significantly facilitated aerobic glycolysis, suggesting that Sox11 enhances glycolysis in OSCC cell lines. Moreover, such an increase was primarily inhibited by LY294002 or MK‐2206, specific PFKM PI3K/Akt signalling pathway inhibitors. This raises the possibility that Sox11 stimulates glycolysis via activating PI3K/Akt signalling. The study also confirmed that Sox11 could regulate the PI3K/AKT pathway in colon cancer cells.^58^

PI3K/Akt signalling is a critical regulator of the proliferation and survival of cells, and altered PI3K/Akt pathway activation is linked to the process of oncogenesis owing to its ability to prevent apoptosis.^59^ PI3K/Akt pathway genes are dysregulated in many oncogenic settings, contributing to the progression of pancreatic, nasal, gastric, and breast cancers.^60^, ^61^, ^62^ Given the close relationship between PI3K/Akt signalling and tumour cell migratory, proliferative, and autophagic activity,^63^, ^64^ studies of the ability of Sox11 to modulate the PI3K/AKT pathway may offer insight into the best approaches to managing OSCC. Besides, the knocking down of Sox11 in OSCC cells suppresses the PI3K/AKT pathway and subsequently suppresses the proliferation and migration of tumour cells. Besides, we also found that the Sox11‐PI3K/AKT pathway is essential for tumour progression in vivo, consistent with clinical data. However, our data preliminarily demonstrated that Sox11 could accelerate the progression of OSCC to a certain extent by activating the PI3K/AKT pathway. According to the literature, there are many downstream pathways regulated by Sox11, such as Wnt/β‐catenin pathway,^65^ SDCCAG8,^48^ HSP90α,^66^ etc. The study about other signalling pathways as potential downstream of Sox11 is the limitation of the current study. In future studies, we will further validate the biological mechanism of Sox11 associated with DNA hypomethylation in OSCC progression.

## 5 CONCLUSIONS

**FIGURE 10:**
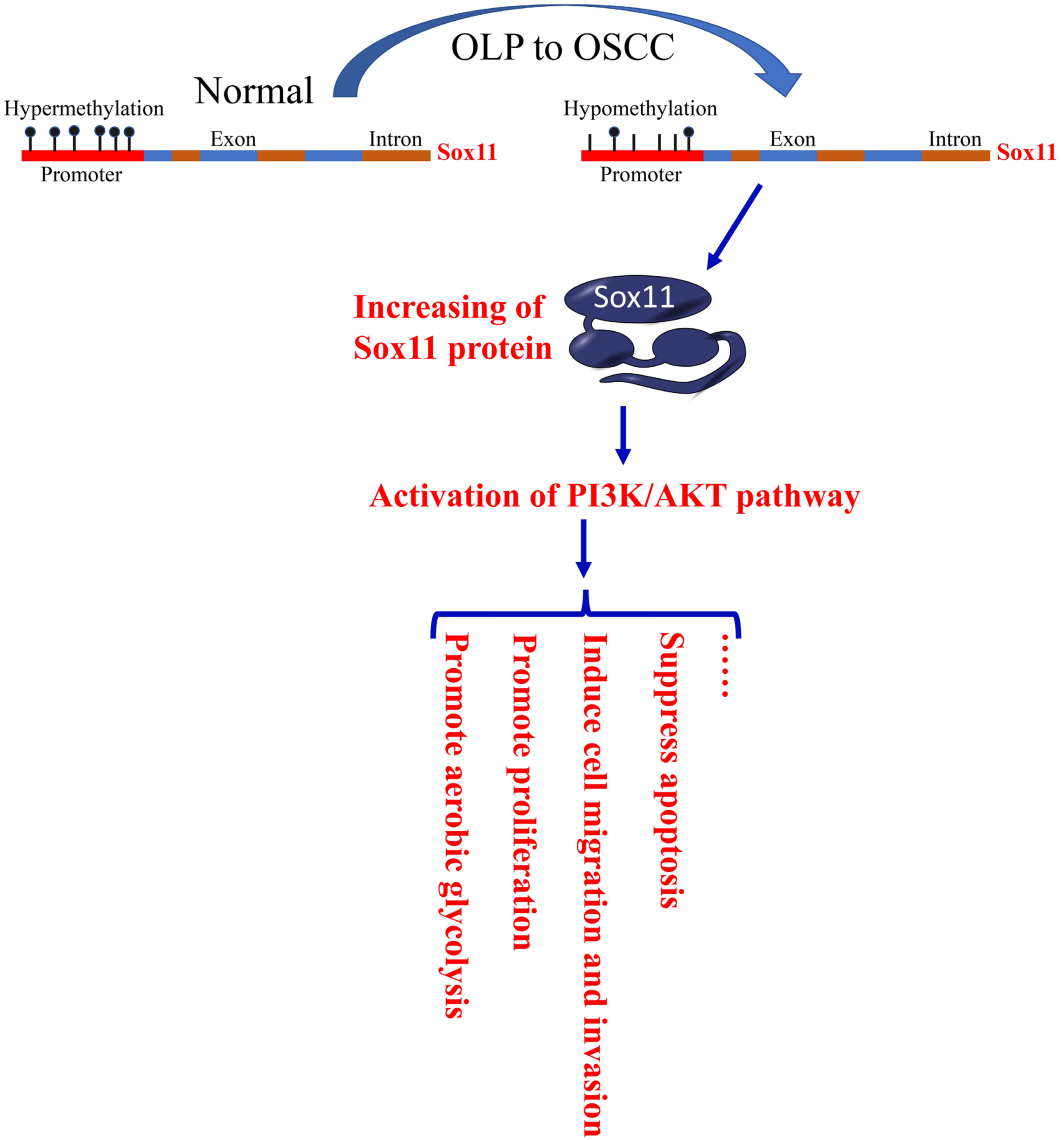
Mechanism of DNA hypomethylation‐associated Sox11‐mediated promotion of glycolysis and oncogenesis through PI3K/AKT signalling in OLP‐associated OSCC.

Our study thus reveals a novel aspect of Sox11 in promoting OSCC by activating PI3K/AKT and glycolysis (Figure 10). The results of this study further demonstrated a central role for the PI3K/AKT pathway in the regulation of Sox11 expression as a mechanism facilitating the maintenance of tumour cell survival and proliferation, providing a foundation to guide future OSCC treatment. In conclusion, these data reveal that promoter hypomethylation drives the upregulation of Sox11 in both OSCC tissues and cell lines. Moreover, Sox11 was shown as a potential oncogenic regulator of tumour development through its ability to promote cellular migration. As such, Sox11 represents a promising biomarker of OSCC and a candidate target for therapeutic intervention, although further work will be critical to validate and expand upon these findings.

## 6 LIMITATIONS OF THE STUDY

Our experimental model system had several limitations in evaluating the DNA hypomethylation‐associated upregulation of Sox11in promoting tumour progression via the PI3K/AKT pathway in OLP‐SOCC. First, as Sox11 is a transcription factor, it would be essential to determine which genes are upregulated due to its (experimental) overexpression or downregulation. A global analysis of gene expression changes allows us to elucidate how PI3K signalling might be stimulated and which other pathways may be activated or inhibited by Sox11 overexpression. Second, knockdown experiments should always be performed with more than one shRNA to avoid unspecific effects. In addition, OSCC has the vital characteristics of tissue heterogeneity and complex tumour microenvironment, which is associated with cancer stem cells (CSCs).^67^ CSCs are a small population of tumour cells with malignant biological behaviours such as promoting tumour malignant proliferation, accelerating tumour metastasis, inducing tumour drug resistance, and causing tumour recurrence, collectively referred to as stemness.^68^ The stemness of CSCs is one of the crucial reasons for the poor prognosis of cancer.^69^ Some studies have successfully isolated OSCC‐CSCs with the help of magnetic bead sorting technology.^70^, ^71^ We will further explore the role of Sox11 in OSCC‐CSCs in the future study.

Moreover, CRISPR/Cas9 has emerged as a powerful gene editing tool with potential in precision medicine.^72^ Jinek et al. demonstrated in 2012 that the CRISPR‐Cas system could produce mature CRISPR RNA (crRNA) and trans‐activating crRNA (tracrRNA), forming a dual RNA hybrid structure.^73^ This double RNA structure can lead the CRISPR‐related protein Cas9 to specific DNA sequences and produce double‐strand breaks in the target DNA.^74^ CRISPR/Cas9 technology has been rapidly promoted and applied in generating animal models, gene function studies, multiplexed mutations, and chromosomal rearrangements. Compared with other gene editing technologies, CRISPR/Cas9 technology is less costly, more efficient, and more straightforward to apply.^75^ In recent years, CRISPR/Cas9 technology has made remarkable progress in many fields. It is of great significance to further study the role of Sox‐11 in OSCC progression using CRISPR/Cas9 technology. In future studies, we will also construct Sox‐11 CRISPR/Cas9 KO plasmids to explore further the role and mechanism of Sox‐11 silencing in OSCC cells.

## AUTHOR CONTRIBUTIONS

**Yi Liu:** Data curation (lead); formal analysis (equal); investigation (equal); project administration (equal); writing – original draft (equal); writing – review and editing (equal). **Peilin Cao:** Investigation (equal); visualization (equal); writing – review and editing (equal). **Li Xiao:** Data curation (equal); visualization (lead); writing – review and editing (equal). **Na Tang:** Methodology (equal); writing – review and editing (equal). **Wei Fei:** Conceptualization (equal); funding acquisition (equal); methodology (equal); validation (equal); writing – review and editing (equal). **Xue Li:** Conceptualization (lead); formal analysis (equal); funding acquisition (equal); supervision (equal); writing – review and editing (equal).

## FUNDING INFORMATION

This study was supported by the National Natural Science Foundation of China (Grant no. 81502362), Science and Technology Foundation of Chengdu, China (Grant no. 2021‐YF05‐00442‐SN), Science and Technology Foundation of Sichuan Province, China (Grant no. 2022YFS0116), Research Fund of Sichuan Academy of Medical Sciences and Sichuan Provincial People's Hospital (2022QN29).

## CONFLICT OF INTEREST STATEMENT

The authors declare that they have no competing interests.

## CONSENT FOR PUBLICATION

Not applicable.

## Supporting information


Figures S1–S3.

## Data Availability

The datasets used and analysed during the current study are available from the corresponding author upon reasonable request.
