# Supplementary material for: Hypomethylation‐associated Sox11 upregulation promotes oncogenesis via the PI3K/AKT pathway in OLP‐associated OSCC
Source: J Cell Mol Med. 2024 Jul 22;28(14):e18556. doi: 10.1111/jcmm.18556 (PMC11263134; doi:10.1111/jcmm.18556)
Supplement: Supplementary file 1 — Figures S1–S3. [file JCMM-28-e18556-s001.pdf]

Supplementary materials

Materials and Methods

Clinical Samples

Between 2010 and 2015, normal oral mucosa (n = 20), OLP (n = 40), and OSCC (n = 120) tissue samples were collected from patients or volunteer participants at the Sichuan Provincial People's Hospital from September 2010 to April 2015 and stored at -80 °C. Of the patients included in this study, 12 underwent progression from OLP to OSCC. OLP was diagnosed as per the 2016 American Academy of Oral and Maxillofacial Pathology criteria [1]. Normal mucosal tissue samples were collected under sterile conditions from areas without evidence of inflammation prior to the development of OLP. All diagnoses were confirmed via pathology analyses, and no patients had undergone chemotherapy or radiotherapy before sample collection. All patients provided written informed consent, and the Ethics Committee of Sichuan Provincial People's Hospital approved this study [Lunshen (Yan) 2022-50].

Cell Culture

Normal human oral epithelial cells (HOECs) and the Cal27, Tca83, SCC-15, SCC-4, SCC-9, and SCC-25, OSCC cell lines were obtained from the American Tissue Culture Collection (ATCC; VA, USA) and Procell Life Science&Technology Co.,Ltd. (Wuhan, China). Cells were cultured in Dulbecco's Modified Eagle's Medium (DMEM; Cat. no. 12800017, Gibco) containing 10% fetal bovine serum (FBS; Solarbio, Beijing, China) and penicillin/streptomycin (Solarbio, Beijing, China) in a humidified 37 °C 5% CO_2 incubator. Cells were passaged when 90-95% confluent.

Transfection and plasmid construction

Lipofectamine 2000 (Invitrogen, Carlsbad, CA, USA) was used to transfect Tca83 and SCC-25 cells with Sox11 shRNA (shRNA-Sox11) or Sox11 overexpression plasmids based on provided directions as previous research [2]. The shSox11 sequence used for this study was: 5’-AATTCAAAAAAGCCTCTACTACAGCTTCAAGAACTCTCTTGAAGTAGCACAACATTCTCCACCCG-3’. The control shRNA (shCTRL) sequence used for the present study was 5’-AATTCAAAAAATTCTCCGAACGTGTCACGTTCTCTTGAATCAGAAAGTTGCTCTTCAGCCG-3’. The pcDNA4.0-Sox11 overexpression plasmid and the empty control pcDNA4.0 plasmid were purchased from Synbio Technologies Co. Ltd. (Suzhou, China). The transfection efficiency of more than 80% indicated successful transfection which confirmed by flow cytometry.

Real-time quantitative polymerase chain reaction (RT-qPCR)

Trizol (Invitrogen) was utilized to extract total RNA from appropriate samples, after which a PrimeScript RT reagent Kit (RR047A, Takara) was used to synthesize cDNA based on provided directions. Fast SYBR Green Mix (Applied biosystems) was then employed for all RT-qPCR analyses together with an ABI PRISM 7300 RT-qPCR instrument (Applied biosystems) using conditions published previously [3]. Relative Sox11 expression was assessed via the $2^{-\Delta\Delta Ct}$ method, with β-actin being used for normalization. Primers were: Sox11-forward:5’- ACGGTCAAGTGCGTGTTTCTG-3’ and Sox11-reverse:5’-TGCTGGTGCGGTGGTTCCTC-3’; β-actin-forward: 5’-CACCATGGATGATGATATCGC-3’ and β-actin-reverse: 5’-CATAGGAATCCTTCTGACCCA-3’.

Bisulfite Detection and Sequencing (BSP)

The Animal Tissues/Cells Genomic DNA Extraction Kit (Solarbio, D1700, China) was used to isolate Genomic DNA from tissues and cells following the manufacturer's instruction. The methylation kit (Zymo Research, Orange, CA, USA) was used for DNA bisulfite conversion [4]. PCR was used to subsequently amplify DNA. Subsequently, amplified DNA product was cloned into pMD19-T vector, The vector after ligation was transformed into DH5α competent cells. Free Endotoxin Plasmid Extraction Mini Kit (Solarbio, D1140, China) was used to purified vectors. The primer sequence for methylated Sox11 was forward: 5'-GGAGAGTAGAGTTTATGTGT-3' and reverse: 5'-CTCCAAACTACTTTACAAAAAT-3'.

CCK-8 assay

The cell after 24 hours transfection was resuspended in complete media and then resuspended and plant onto 96-well plates (2×10^3/well in 100 μL) and cultured for 0, 24, 48, or 72 hours, 10 μL of the CCK-8 solution (Sigma-Aldrich; Merck KGaA) was added in each well. Following a 2 h incubation, the absorbance was then measured with a Tecan microplate reader (Tecan Group, Ltd.).

EdU uptake assay

For EdU incorporation staining, a YF®594 Click-iT EdU Imaging Kit (YEASEN Biotech Co. Ltd, Shanghai, China) was used according to the product specification. Tca83 and SCC-25 cells with different treatment were seeded in 96-well plates at a density of 5×10^4 cells/well for 24 h. The cell nuclei were stained using 4′,6-diamidino-2-phenylindole (DAPI, Biosharp, BL105B, Shanghai, China). The positive cells (red fluorescence) were visualized by fluorescence microscopy, imaged, and analyzed using ImageJ software (Version 1.5.3). The experiment was replicated three times, and the typical result

was shown.

Flow Cytometry

At 48 h post-transfection, cells from 6-well plates were collected with EDTA-free trypsin, rinsed with cold phosphate-buffered saline (PBS; Sigma Aldrich, St. Louis, MO, USA), centrifuged, and stained with an annexin V-FITC apoptosis detection kit (BioLegend, Inc.). Cells were then analyzed with a FACSCalibur Flow Cytometry instrument (BD Biosciences).

Transwell assays

Cellular migration and invasivity were assessed using 24-well Transwell inserts (8-μm-pore size, NY, USA) that either were not or were coated with Matrigel, respectively. Briefly, 5×10^4 transfected cells were added to the upper compartment in 300 uL of serum-free media, with media supplemented with 10% FBS being added in the lower compartment. Following a 24 h incubation, cells that had entered the lower chamber were fixed using 4% paraformaldehyde (PFA; Santa Cruz, CA, USA), stained using 0.5% crystal violet, and imaged with a light microscope (BX51, Olympus Corporation; 200x), with cells in 5 random fields of view being quantified for analysis.

Western blotting

Radioimmunoprecipitation assay (RIPA) buffer (Beyotime, China) containing 1% protease inhibitor cocktail (KeyGEN BioTECH; Nanjing, China) and 1% phenylmethanesulfonyl fluoride (PMSF; Sigma, Cat #P7626) was employed to lyse cells, after which protein levels were assessed using a Bicinchoninic acid (BCA) Protein Assay Kit (KeyGEN BioTECH). Equal amounts of protein from each sample were

separated via 10% SDS-PAGE, transferred onto PVDF membranes, and blots were blocked for 2 h at room temperature using 5% non-fat milk. Primary antibodies specific for Sox11 (1:1000, cat. No. ab134107, Abcam), p-PI3K-p85 (1:1000, cat. No. ab182651; Abcam), PI3K-p85 (1:1000, cat. No. ab191606; Abcam), pAKT (1:1000, cat. No. ab38449; Abcam), AKT (1:1000, cat. No. ab8245; Abcam) or GAPDH (1:1000, cat. No. ab6046; Abcam) were then used to probe blots overnight at 4℃. After blots were washed one time, they were incubated with HRP-conjugated goat anti-rabbit IgG (1:5000; cat. No. ab205719; Abcam) or goat anti-mouse IgG (1:10000; cat. No. ab205719; Abcam). Protein bands were then detected using an enhanced chemiluminescence (ECL) detection reagent (GE Healthcare Life Sciences, UK).

Glucose uptake and lactate production assay

For the glucose uptake assay, Cells were pre-grown in six-well plates, after which cells were first incubated with low concentrations of glucose for four hours, followed by thirty minutes in glucose-free medium, after which 1.5 μg/ mL 2NBDG (cat. no. K682-50; BioVision, Inc.) was added for thirty minutes of treatment. Afterwards, cell fluorescence levels were assessed using flow cytometry. For the lactate production assay, cells were grown in six-well plates and after 24 hours the medium was changed, and 5 mM glucose was added and incubated for 15 hours. Finally evaluate of lactate concentration in the medium using lactate colorimetric assay kit (Biovision, K607-100).

Intracellular ATP assay

ATP assay kit (Beyotime, S0026) was used to analysis ATP level. Briefly, Cells were grown in 12-well plates and after 24 hours, harvested cells were lysed with 200 μL lysis solution and the ATP content of

the supernatant was assessed after centrifugation use Multiscan Spectrum (Luminescence).

Seahorse assay

We assessed the oxidative phosphorylation as well as glycolysis level of OSCC cells after Sox11 overexpression using the XF96 extracellular flux analyzer (Seahorse Bioscience), The cells were pre-grown in 12-well plates and pre-changed to experimental medium one hour before the experiment. After assessing baseline, various metabolic inhibitors were added successively to assess extracellular acidification rate (ECAR) and oxygen consumption rate (OCR).

Mice experiments

The animal experiment was permitted by the Institutional Animal Care and Use Committee of Sichuan Provincial People's Hospital and performed in accordance with the National Institutes of Health Guide for the Care and Use of Laboratory Animals. All animal experiments were placed in Specific-pathogen-free (SPF) grade Animal Laboratory at Animal Laboratory Center of Sichuan Provincial People's Hospital.

Four-week-old athymic BALB/c nude mice were purchased from SLAC Laboratory Animal Co. (Shanghai, China) and were kept in pathogen free environment at Animal Laboratory Center of Sichuan Provincial People's Hospital and housed under standard 12 h/12 h light/dark conditions. During the experiment, 1×10^7 SCC-25 wild type or Sox11 up-regulated SCC-25 or Sox11 down-regulated SCC-25 or negative control shRNA or vector expression SCC-25 was injected into nude mice subcutaneously. Tumor volumes were measured every 3 days. After 28 days mice were euthanized with 1% sodium pentobarbital (i.p. injection) and were decapitated, then tumors were obtained for further assay. Besides,

primary tumor cells were isolated from xenograft tissues and then used for metabolic measurements.

Immunohistochemistry (IHC)

IHC staining was conducted as in prior reports [5]. Paraffin sectioning was performed in a sequence involving de-paraffination, antigen retrieving, endogenous-peroxidase inactivation, block-step using goat serum (Gibco™, USA), followed by incubation with Ki-67 (Abcam™; #ab15580; 1:400 dilution) primary antibody overnight at 4℃ and secondary antibody (abcam; #ab205718; 1:1000 dilution) for 1 h at room temperature. Tissue segments were finally subjected to visualization with a diaminobenzidine (DAB) Kit (Invitrogen) and imaging under a phase-contrast microscope (Leica™, Cat. #DMI 1).

Terminal deoxynucleotidyl transferase-mediated dUTP nick-endlabeling (TUNEL) assay

Single-step TUNEL kit (C1089, Beyotime Institute) was used as described before [6]. Briefly, tissue segments (3 μm) from individual cohorts were de-waxed. Post- protease K incubating (no DNase, 20 μg/mL) for 0.5 hours at 37℃ and a PBS-rinse, segments were placed into incubation again, with 50 μL TUNEL solution for one hour at 37℃ in darkness. Consequently, stained-segments were PBS-rinsed and visualized through fluorescence confocal microscopy (Zeiss ™ LSM710®, Germany). Quantification of TUNEL positive cellular population was determined across ten randomized fields over six segments through Image J ® (Bio-Rad Laboratories™, USA) for comparative analyses.

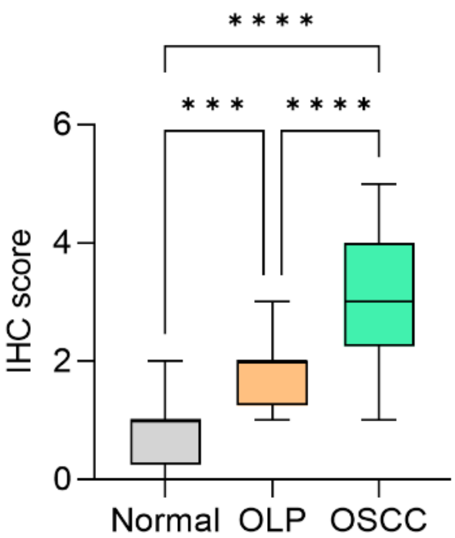

Figure S1. The results of IHC have been quantitatively analyzed. **** $P < 0.0001$.

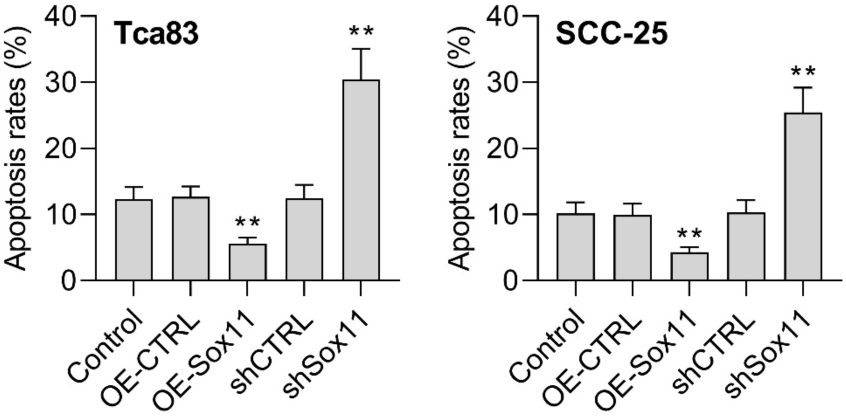

Figure S2. quantification of cell apoptosis. ** $P < 0.01$.

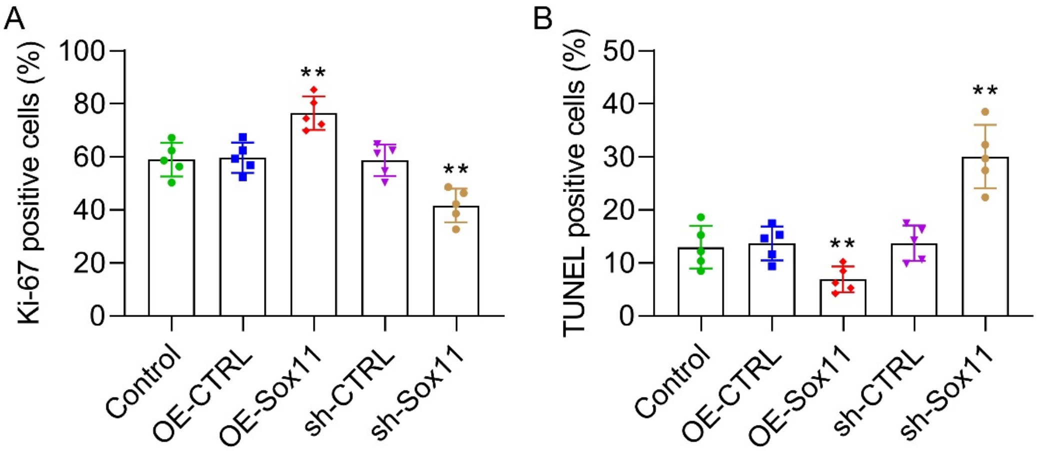

Figure S3. Quantification of Ki-67 positive cells and TUNEL positive cells. (A) Ki-67 positive cells

number was shown in CTRL, OE-CTRL, OE- Sox11, shCTRL and sh-Sox11 cohorts, respectively. (B) Quantification of TUNEL positive cells in CTRL, OE-NC, OE- Sox11, shCTRL, and sh-Sox11 cohorts in-tumor.

Reference

1. Cheng YS, Gould A, Kurago Z, Fantasia J, Muller S. Diagnosis of oral lichen planus: a position paper of the American Academy of Oral and Maxillofacial Pathology. *Oral Surg Oral Med Oral Pathol Oral Radiol*. Sep 2016;122(3):332-54. https://doi.org10.1016/j.oooo.2016.05.004.
2. Xiao L, Li X, Cao P, et al. Interleukin-6 mediated inflammasome activation promotes oral squamous cell carcinoma progression via JAK2/STAT3/Sox4/NLRP3 signaling pathway. *Journal of experimental & clinical cancer research : CR*. May 5 2022;41(1):166. https://doi.org10.1186/s13046-022-02376-4.
3. Barbut F, Monot M, Rousseau A, et al. Rapid diagnosis of Clostridium difficile infection by multiplex real-time PCR. *Eur J Clin Microbiol Infect Dis*. Oct 2011;30(10):1279-85. https://doi.org10.1007/s10096-011-1224-z.
4. Liu X, Tang X, Zhang S, et al. Methylation and Expression of Retinoblastoma and Transforming Growth Factor-beta1 Genes in Epstein-Barr Virus-Associated and -Negative Gastric Carcinomas. *Gastroenterol Res Pract*. 2012;2012:906017. https://doi.org10.1155/2012/906017.
5. Liu J, Yang Q, Sun H, Wang X, Saiyin H, Zhang H. The circ-AMOTL1/ENO1 Axis Implicated in the Tumorigenesis of OLP-Associated Oral Squamous Cell Carcinoma. *Cancer Manag Res*. 2020;12:7219-7230. https://doi.org10.2147/CMAR.S251348.
6. Wang XW, Tian RM, Yang YQ, et al. Triptriolide antagonizes triptolide-induced nephrocyte apoptosis via inhibiting oxidative stress in vitro and in vivo. *Biomed Pharmacother*. Oct 2019;118:109232. https://doi.org10.1016/j.biopha.2019.109232.
